# Hope in working-age adults with irreversible vision loss: a scoping review

**DOI:** 10.1590/1980-220X-REEUSP-2026-0181en

**Published:** 2026-08-14

**Authors:** Cátia Isabel Marranita dos Santos Duarte, Ana Isabel Batalha Bicho, Mónica Figueiredo da Silva Bento, Amélia Simões Figueiredo, Ana Resende

**Affiliations:** 1 Universidade Católica Portuguesa, Faculdade de Ciências da Saúde e Enfermagem, Lisboa, Portugal.; 2 Unidade Local de Saúde de Almada Seixal, Almada, Setúbal, Portugal.

**Keywords:** Adult, Blindness, Nursing, Hope, Coping Skills

## Abstract

**Objective:**

To map existing scientific evidence on hope in working-age adults facing irreversible vision loss by identifying concepts, approaches, and gaps in the literature.

**Method:**

A scoping review was conducted in accordance with the JBI methodology and the PRISMA-ScR guidelines using international databases and grey literature, covering studies published between 2000 and 2025. The identified studies underwent a process of identification, selection, data extraction, and thematic synthesis by two independent reviewers, with the support of NVivo® software.

**Results:**

Of the 261 studies identified through the literature search, six met the inclusion criteria. Analysis of the included studies allowed the identification of four main topics: (1) Concept and sources of hope; (2) Hope as a coping mechanism; (3) Psychosocial adjustment; and (4) Implications for clinical practice. Hope emerged as a multidimensional and dynamic phenomenon, with a direct impact on adaptation, quality of life, and the meaning attributed to the experience of vision loss.

**Conclusion:**

Hope plays a fundamental role in the adaptation process to vision loss among working-age adults, with important implications for the development of person-centered nursing practices. Further research in this field will contribute to strengthening the theoretical knowledge of nursing science and guiding clinical interventions aimed at promoting hope.

## INTRODUCTION

Irreversible vision loss is both a traumatic and profoundly challenging event, particularly when it occurs in workingage individuals, a stage of life in which personal and social identity is strongly grounded in autonomy, productivity, and the performance of family, professional, and social roles. When experienced during this stage of the life cycle, vision loss constitutes a complex health transition, triggering profound repercussions on identity, life projects, and the ways individuals relate to themselves, to others, and to the world.

The age range between 18 and 65 years was defined because it corresponds to the period commonly associated with working age, during which adult identity is strongly related to autonomy, participation in the workforce, and the performance of family, professional, and social roles. At this stage of the life cycle, work and social roles have particular importance in the construction of the self, the sense of competence, and psychological well-being^(1,2)^. Thus, irreversible vision loss may represent a significant disruption to life projects, social participation, and occupational integration, making it pertinent to examine hope as an adaptive resource throughout the transition process experienced by this population^(1,2,3)^.

Vision plays a fundamental role in the construction of the self and in interaction with the surrounding context, as its loss requires a substantial reorganization of the sense of continuity, expectations regarding the future, and the reference points that sustain everyday life. Recent evidence demonstrates that vision loss directly affects quality of life, future plans, and psychological well-being, representing a considerable emotional and psychosocial burden for affected individuals and requiring complex adaptive processes that extend far beyond functional impairment^(4)^.

Vision loss is also associated with a substantial psychological impact, triggering a range of emotional and behavioral responses that require continuous adaptive adjustment, with implications for the mental health and psychological well-being of affected individuals^(4)^.

Qualitative studies indicate that severe vision loss profoundly affects everyday life, social participation, and the perception of life’s meaning, suggesting that adaptation encompasses multiple domains of the human experience^(5)^.

Reviews addressing well-being among adults with visual impairment highlight that psychosocial adaptation depends on interconnected factors, including personal identity, social support, and a sense of coherence in life^(6)^.

Within this context, hope emerges not merely as an emotional state but as a dynamic process of adaptation that enables individuals to reconstruct meaning, redefine goals, and identify new forms of participation and purpose. Despite its recognized importance in the contexts of chronic illness and health transitions, the evidence regarding hope among workingage adults with irreversible vision loss remains fragmented and insufficiently systematized^(7,8)^.

Although the ophthalmology literature has progressively emphasized the subjective and psychosocial dimensions of vision loss^(9,10)^, the understanding of hope as a phenomenon associated with lived experience and adaptation processes remains insufficiently systematized^(3,8,10)^. Therefore, it is important to deepen knowledge of how hope manifests in working-age individuals with irreversible vision loss, particularly within the context of transition, psychosocial adjustment, and the reconstruction of meaning in response to their new life condition.

Accordingly, this scoping review aims to map the existing literature on this phenomenon, thereby contributing to the development of more humanized, person-centered care practices.

## METHOD

### Study Design

The search was conducted in November 2025, in accordance with the JBI Manual and the Preferred Reporting Items for Systematic Reviews and Meta-Analyses extension for Scoping Reviews (PRISMA-ScR) guidelines. The complete search strategy, including the combinations of search terms used in each database, is available in the protocol previously registered on the Open Science Framework^(11)^, DOI 10.17605/OSF.IO/ GCXEV, in accordance with the principles of transparency and reproducibility. The research question guiding this review was: How does hope influence the transition process and the coping strategies of working-age individuals experiencing irreversible vision loss? The methodological framework was structured according to the PCC (Population, Concept, and Context)^(11)^.

### Population and Selection Criteria

The population included working-age adults, aged between 18 and 65 years, with irreversible vision loss, regardless of its etiology. The central concept investigated was hope as a psychological, social, and spiritual phenomenon associated with the processes of adaptation and transition. The context encompassed all settings, including clinical, social, and community perspectives.

Studies published between 2000 and 2025 in Portuguese, English, or Spanish were included. Studies addressing reversible vision loss or blindness diagnosed before the age of 18 years or after the age of 65 years were excluded.

### Data Collection

The literature search was conducted in the MEDLINE Complete, Scopus, and Web of Science databases, as well as in the grey literature through the ProQuest Dissertations & Theses Citation Index. The search strategies combined free-text terms and Medical Subject Headings related to hope, vision loss, and working age, adapted for each database.

The identified records were imported into the Rayyan platform, where duplicates were removed and title and abstract screening was independently performed by two reviewers. Potentially eligible articles were subsequently assessed in full text, maintaining the duplicate review process, with a third reviewer consulted whenever disagreements arose.

### Data Analysis and Processing

Data were analyzed using descriptive and thematic approaches with the support of NVivo^®^ software (Lumivero version 14), and the findings were organized into topics and subtopics.

Data analysis followed a predominantly inductive thematic approach, allowing the identification of patterns, topics, and meanings emerging from the included evidence. The analytical process involved an in-depth reading of the studies, coding of relevant excerpts, and subsequent grouping into thematic categories and subtopics related to hope, coping strategies, psychosocial adjustment, and implications for clinical practice.

NVivo^®^ software (Lumivero version 14) was used to support the organization, management, and coding of the qualitative data extracted from the studies, facilitating the systematic organization of the findings and the identification of relationships among emerging topics.

Variables related to the methodological characteristics of the studies (author, year, country, study design, population, context, and objectives) were extracted, together with data concerning the conceptualization of hope, facilitating and hindering factors, adaptive mechanisms, the psychosocial impact of vision loss, and implications for nursing practice.

### Ethical Aspects

The ethical principles governing scientific research were respected, particularly regarding integrity, methodological rigor, transparency throughout the review process, and the appropriate citation of all sources used, in accordance with international guidelines for literature reviews.

## RESULTS

**Figure 1:**
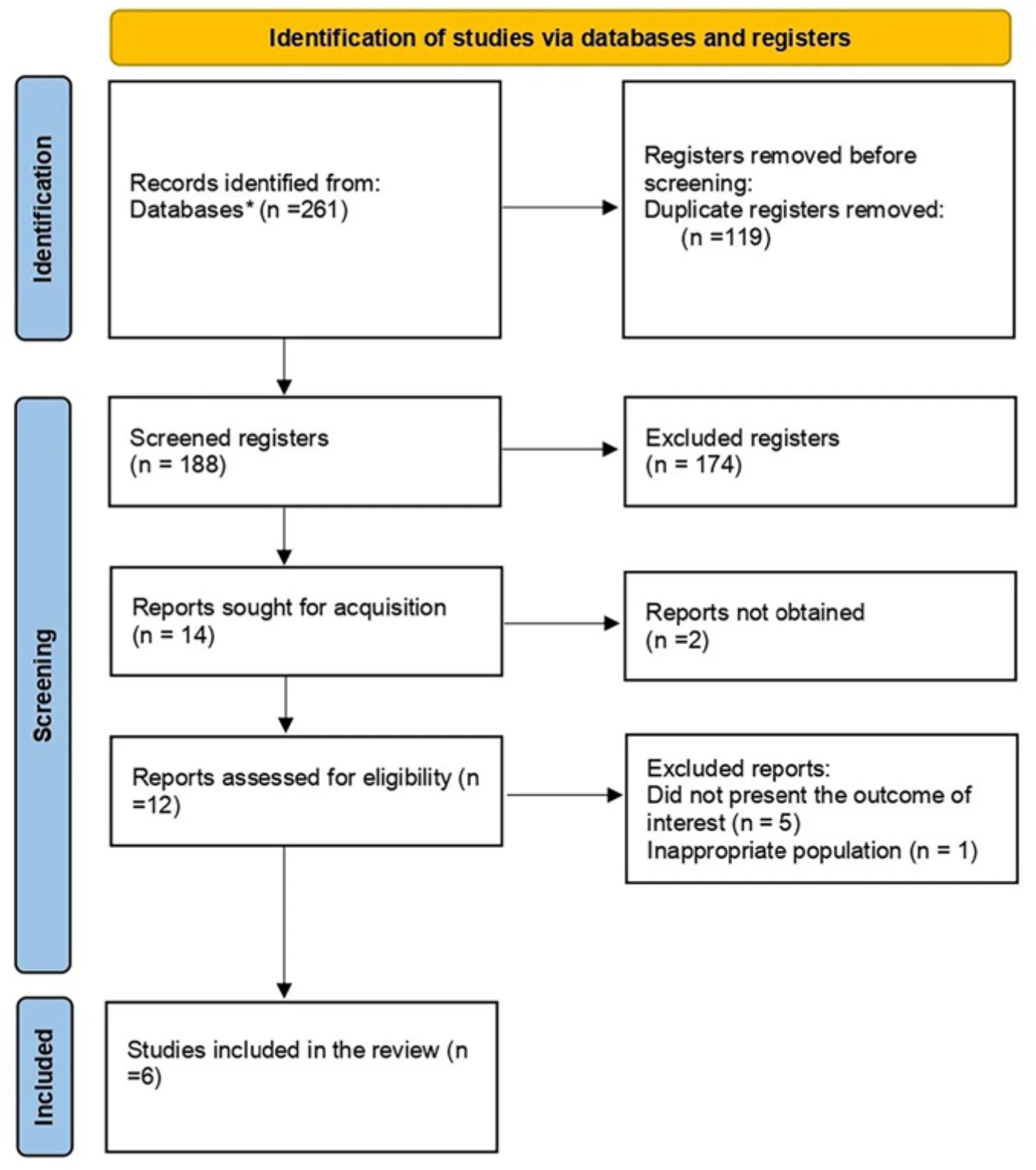
Preferred Reporting Items for Systematic reviews and Meta-Analyses extension for Scoping Reviews flowchart diagram.

The literature search identified 261 records. After duplicate removal and the application of the inclusion and exclusion criteria, six studies were included in this scoping review. The processes of identification, screening, eligibility assessment, and study inclusion are illustrated in the PRISMA-ScR flow diagram (Figure 1).

The included studies were published between 2008 and 2022. Of the six studies included, two employed a qualitative methodology (33.3%), three adopted a quantitative methodology (50.0%), and one consisted of a narrative/reflective report (16.7%). Concerning geographical distribution, studies conducted in the United States of America (50.0%) and the United Kingdom (33.3%) predominated. The studies primarily addressed topics such as hope, social support, quality of life, lived experience, and psychosocial adjustment associated with irreversible vision loss.

Methodological heterogeneity was observed, encompassing cross-sectional quantitative studies, exploratory qualitative studies, and narrative reports, mainly focusing on the subjective experience of vision loss, hope, social support, quality of life, and psychosocial adaptation processes.

Chart 1 summarizes the main characteristics of the included studies, namely the authors, year of publication, country of origin, study design, population, objectives, and principal findings.

**Chart 1:** Summary of the main characteristics of the included studies – Lisbon, Portugal, 2026.

Article title	Authors/year	Origin country	Method	Population	Objective	Mais results
Disability Self-Worth and Positive Personal Meaning in Disability: Correlates of Hope Among U.S. Residents With Physical Disabilities^(7)^	Zapata MA (2022)	USA	Cross-sectional quantitative	Adults with physical and sensory disabilities	Analyze the relationship between disability identity, personal meaning, selfesteem, and hope.	Personal self-worth and the positive meaning attributed to disability were significantly associated with higher levels of hope.
Hope and Social Support in Adults Who Are Legally Blind at a Training Center^(8)^	Singletary C, Goodwyn MA, Carter AP (2009)	USA	Cross-sectional correlational quantitative	Legally blind adults attending a rehabilitation center	Examine the relationship between hope and social support.	Hope was positively associated with perceived social support, including support from friends, family, and significant others.
Impact of Low Vision on Well-Being in 10 European Countries^(12)^	Mojon-Azzi SM, Sousa-Poza A, Mojon DS (2008)	Ten European countries	Observational quantitative	Individuals aged ≥50 years	Analyze the association between low vision and wellbeing.	Low vision was associated with poorer emotional well-being, lower hope for the future, sadness, fatigue, irritability, and difficulties with concentration.
Information Gives Hope^(10)^	Lesselroth M (2019)	USA	Narrative/ reflective report	Adult with glaucoma	Describe the experience of receiving the diagnosis and the role of information.	Clear information, accessible resources, and peer support promoted hope, a sense of control, and adaptation to the diagnosis.
Seeing Beyond Anatomy: Quality of Life with Geographic Atrophy^(9)^	Caswell D, Caswell W, Carlton J (2021)	Canada/ United Kingdom	Qualitative study incorporating the perspectives of patients and caregivers	Individual with geographic atrophy and an informal caregiver	Explore the impact of geographic atrophy on quality of life.	Vision loss affected autonomy, hobbies, social relationships, and quality of life. In this context, information, support, and assistive technologies emerged as important resources.
Experiencing Motherhood as a Blind Mother in the Greater Accra Region of Ghana; a Qualitative Study^(3)^	Acheampong AK, Marfo M, Aziato L (2022)	Ghana	Exploratory descriptive qualitative	Nineteen blind mothers	Explore the experience of motherhood among blind women.	Difficulties related to the maternal role, discrimination, psychological distress, and coping strategies centered on prayer, self-motivation, and hope in their children emerged.

Legend: USA – United States of America.

The thematic analysis allowed the identification of four main topics: (1) Concept and sources of hope; (2) Hope as a coping mechanism; (3) Psychosocial adjustment; and (4) Implications and gaps for practice.

Chart 2 presents a synthesis of the findings organized according to the identified topics, including representative excerpts and their respective source studies.

**Chart 2:** Summary of the findings according to the identified topics – Lisbon, Portugal, 2026.

Main topics	Topic and subtopics	Literal excerpts (en)
1. – Concept and sources of hope	1 – Concept and sources of hope	“*Hope is a process of thinking about one’s goals...*”^(7)^ “*Cognitive motivational system involving both goal-directed motivation and consideration of means to achieve goals.*”^(7)^ “*Hope has been associated with various positive psychological, physical health, and academic outcomes.*”^(7)^ “*Self-worth and personal meaning predicted higher hope.*”^(7)^ *“Hope is a process of thinking about one’s goals that involves: (a) the motivation to move toward those goals(agency); and (b) the consideration of means to achieve those goals*.”^(7)^ “*Knowing about the current evidence... helps patients stay positive.*”^(10)^
	1.1 – Factors promoting hope	“*High levels of hope were tied to strong support networks*.”^(8)^ *“The length of time spent at training centers would be associated with levels of self-esteem and hope*.”^(8)^ *“Positive relationships that were found between hope and perceived social support*.”^(8)^ “*Magnifying devices can reduce the degree of handicap.*” ^(8)^ “*Prayer was done in such a way that, they could talk to God at different times.*”^(3)^ “*It would have helped to have a list of resources to take home after the diagnosis.*”^(10)^ *“Having access to information* (...) *and knowing what developments are being worked on gives me hope and peace of mind.*”^(10)^
	1.2 – Factors hindering hope	“*Patients lack information around their expected prognosis...*”^(9)^
2. – Hope as a coping mechanism	2.1 – Active or proactive coping	“*I took matters into my own hands... started my own support group.*”^(10)^
	2.2 – Emotion-focused or spiritual coping	“*Prayer was the safe haven...*”^(3)^ “*Participants* (...) *coped with their challenges with prayer, hope in their children and self-motivation.*”^(3)^ *“I hope I’m at the plateau of visual loss, but I don’t know*.”^(9)^
	2.3 – Relationship with personal identity	“*Hope has been positively associated with self-esteem...*”^(7)^
	2.4 – Resilience and self-efficacy	“*Developing strength in adversity...*”^(7)^ “*The long-term blindness of these individuals may have bolstered their self-acceptance and adjustment to blindness.*”^(8)^
3. – Psychosocial adjustment	3.1 – Social and interpersonal support	“*Hope was tied to strong social support networks...*”^(8)^ “*Their children were their source of hope...*”^(3)^
	3.2 – Quality of life and well-being	“*Persons with low vision... more likely to wish for death.*”^(9)^
	3.3 – Psychological outcomes	“*Identifying and encouraging development of strengths...*”^(7)^
4. – Implications and gaps for practice	4.1 – Intervention suggestions for practice	“*Let patients know there is hope... make them aware of resources*.”^(10)^ “*Make patients aware of the resources available to them, including online support groups.*”^(10)^ *“Knowing about the current evidence on disease progression and sharing it with patients can help them stay positive.”* ^(10)^
	4.2 – Gaps in the literature	“*Research has yet to explore disability self-worth...*”^(7)^

**Figure 2:**
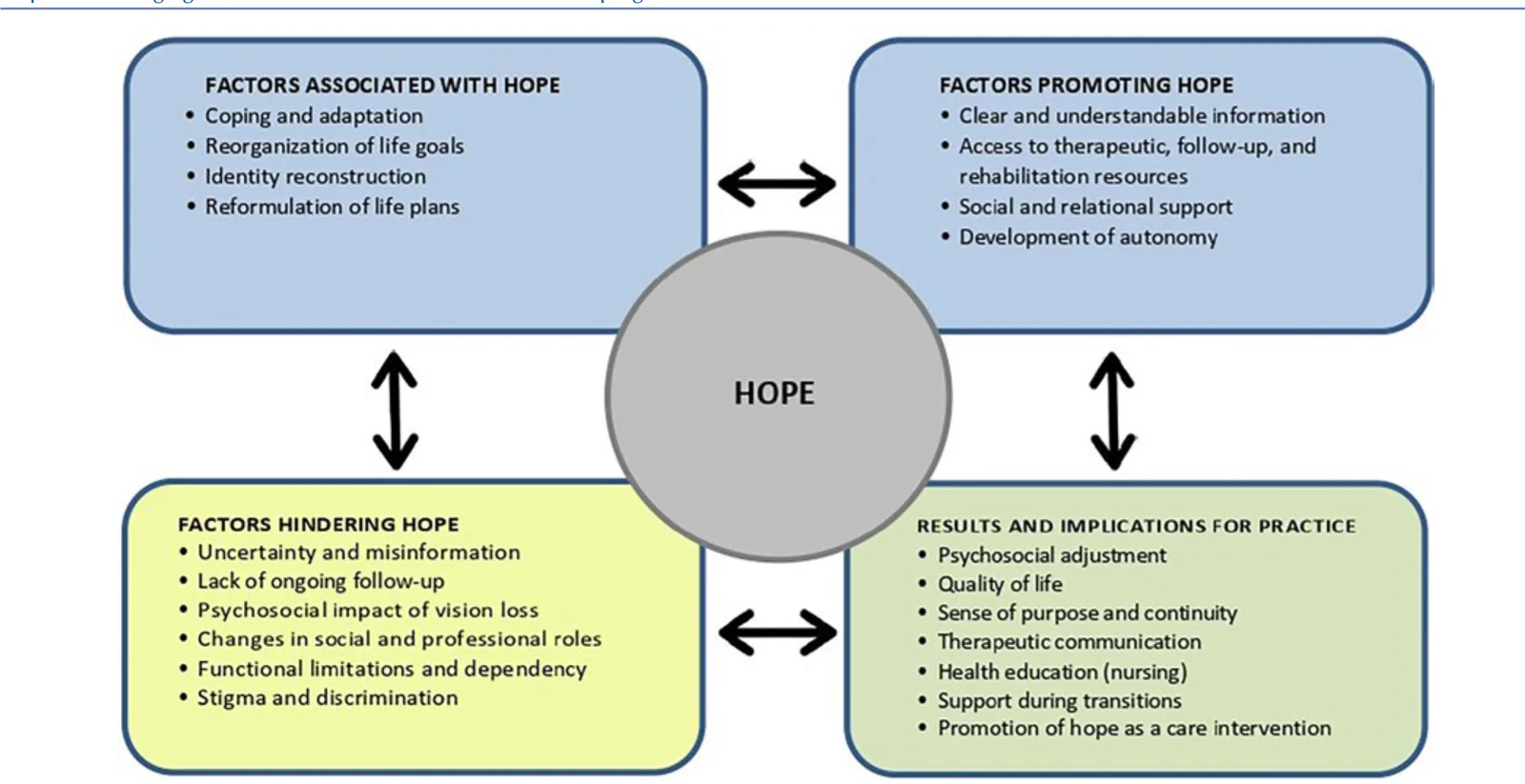
Summary of the findings.

Across the analyzed studies, hope emerged as a multidimensional resource integrating cognitive, relational, and existential components. The thematic analysis demonstrated that access to information, meaningful social support, and the reconstruction of personal meaning function as convergent factors promoting hope, positively influencing psychosocial adjustment and coping strategies in response to irreversible vision loss^(3,8,9,10)^, as illustrated in Figure 2.

## DISCUSSION

The interpretive approach adopted in this study is grounded in the understanding of hope as a complex phenomenon within the context of health processes, integrating an individual’s experience, transition processes, and care contexts. This perspective makes it possible to analyze, in an integrated manner, the individual and relational dimensions that influence hope, thereby contributing to the understanding of human responses and to the development of nursing interventions that are better aligned with clinical practice. By highlighting hope as a relational and context-dependent adaptive resource, the findings of this scoping review reinforce the relevance of perspectives that move beyond a biomedical view of vision loss, emphasizing the subjective, social, and existential dimensions that influence biopsychosocial adjustment.

In light of the included studies, hope emerges as a complex construct, predominantly understood as a goal-directed cognitive-motivational process rather than as an isolated emotional state^(3,7,8,12)^.

The analyzed evidence supports hope as the ability to mobilize motivation toward meaningful goals while simultaneously identifying concrete pathways to achieve them, even in contexts of adversity^(7,8,12)^.

This conceptualization is supported by empirical findings demonstrating consistent associations between higher levels of hope and positive indicators of psychological adjustment, particularly self-esteem, personal self-worth, and the meaning attributed to lived experience, reinforcing the role of hope as a central adaptive resource^(3,7,8)^.

Across the studies analyzed, hope assumes a strongly relational and contextual nature, being influenced by factors that may either strengthen or undermine it^(3,8,9,10,13)^.

Access to clear, understandable, and timely information regarding the diagnosis, disease progression, and available resources consistently emerged as a facilitator of hope, as it enables individuals to anticipate changes, manage expectations, and reduce uncertainty about the future^(3,8,9,13)^.

Likewise, the presence of meaningful social support networks, including peers, healthcare professionals, and educational or rehabilitation settings, was associated with higher levels of hope, facilitating coping mechanisms and psychosocial adjustment^(3,8,10,13)^.

In contrast, contexts characterized by inadequate information, lack of follow-up, stigma, or negative societal attitudes were associated with diminished hope, intensifying feelings of uncertainty, isolation, and loss of control^(3,10,14)^.

Within the specific context of irreversible vision loss during working age, hope emerges as a central phenomenon in the adaptation process, constituting a relational and dynamic adaptive resource^(7,8,9,10,12)^.

Within this framework, hope is associated with self-esteem, personal self-worth, the perceived ability to reorganize goals and daily routines, the construction of meaning in response to vision loss (including identity reconstruction and the redefinition of socially valued roles), and perceived social support^(7,8,10,12)^.

Thus, hope does not manifest as the denial of the irreversible nature of the condition but rather as the ability to reorganize life goals, identify feasible solutions, and attribute continuity and meaning to one’s experience despite progressive functional limitations, reflecting the multidimensional, dynamic, and context-dependent nature of psychosocial adjustment in this setting^(3,8,9,10)^.

The analysis of quality of life in the context of irreversible vision loss suggests that adaptation extends beyond anatomical or functional dimensions, arising primarily from the way individuals reinterpret their lived experiences, reconstruct their identities, and redefine what it means to live well despite the progression of the condition. From this perspective, hope is implicitly expressed as a sense of continuity, purpose, and value attributed to life, even in the presence of progressive and irreversible functional limitations^(3,9)^.

The analyzed studies demonstrate that the information provided to patients plays a fundamental role in building and sustaining hope, extending beyond an instrumental function to become a central element in promoting a sense of control, predictability, and the ability to anticipate the future. Access to clear, understandable, and person-tailored information, particularly regarding prognosis, the progression of vision loss, and the available therapeutic, rehabilitation, and social resources, consistently emerges as a factor that facilitates psychological adjustment. This contributes to reducing uncertainty, fear, and feelings of hopelessness while supporting more adaptive coping processes and the reorganization of everyday life^(3,7,8,9,10)^.

Despite the relevance of this phenomenon, the literature reveals significant gaps, particularly regarding the development and assessment of structured hope-promoting interventions, especially within nursing. In this context, nursing care integrated with therapeutic communication provides an opportunity to foster hope through health education and continuous follow-up, transforming information into meaningful knowledge, promoting individuals’ active participation, and supporting more autonomous, dignified, and psychosocially adjusted adaptation processes in response to irreversible vision loss during working age.

### Study Limitations

Despite the methodological rigor adopted, this scoping review has some limitations that should be acknowledged. The limited number of studies included reflects both the specificity of the phenomenon investigated and the scarcity of published research directly and systematically addressing hope in the context of irreversible vision loss among working-age adults, which justified extending the search period to the previous 25 years. Moreover, the available studies were conducted in heterogeneous contexts with respect to participants’ age, the etiology of visual impairment, and sociocultural settings.

The predominance of qualitative and exploratory approaches, frequently based on small samples, limits the transferability of the findings. Furthermore, the conceptual diversity surrounding the definition of hope—variously described as a cognitive-motivational resource or as an existential or spiritual experience—makes comparisons across studies difficult and hinders the consolidation of a unified theoretical framework.

There is also limited exploration of the temporal dimension of hope, with few studies examining its evolution throughout the adaptation process following vision loss. Finally, the absence of intervention studies limits the applicability of these findings to evidence-based clinical practice.

### Suggestions for Improvement and Future Research

The findings of this scoping review reinforce the relevance of conducting in-depth research on the transition process experienced by working-age adults facing vision loss. The relationship among hope, meaning attributed to the experience, coping strategies, identity reconstruction, and relational support identified in the reviewed literature highlights the need to understand this phenomenon as a dynamic and contextualized process, integrating the biological, psychological, social, and spiritual dimensions of lived experience. These findings support the adoption of an interpretive qualitative approach, such as Constructivist Grounded Theory, as a methodology capable of capturing the complexity of the transition process and giving voice to individuals in constructing the meanings associated with vision loss.

Future studies should also prioritize longitudinal designs to better understand the evolution of hope from the time of diagnosis through more advanced stages of adaptation and identity reconstruction, further exploring hope as a dynamic process in the context of irreversible vision loss.

The validity of instruments sensitive to the subjective experience of hope among individuals with visual impairment is also recommended as another important research priority.

In the field of clinical practice, intervention studies are needed to assess structured hope-promoting strategies through educational programs, transition-focused nursing follow-up, and facilitated access to information and support networks, with the aim of providing more humanized and patient-centered care^(3,7,8,9,10,11,12)^.

## CONCLUSION

This scoping review consistently demonstrates hope as a central adaptive resource in the transition process experienced by working-age individuals facing irreversible vision loss. The findings indicate that hope constitutes a dynamic, relational, and contextual phenomenon that is closely associated with selfesteem, personal meaning, social support, and access to clear and relevant information, playing a decisive role in psychosocial adjustment, the reorganization of life projects, and identity reconstruction.

Despite these contributions, the literature remains limited and fragmented, characterized by the predominance of exploratory approaches, the scarcity of longitudinal studies, and the absence of structured interventions aimed at promoting hope, particularly within nursing. These findings reinforce the need for further research and for the development of integrative conceptual models capable of supporting evidence-based clinical practice.

Within this context, nursing emerges as a discipline with a key role in fostering hope through person-centered practices that emphasize therapeutic communication, health education, support throughout the transition process, and facilitation of access to resources and support networks, thereby contributing to more humanized, integrated, and experiencesensitive care, with positive impacts on dignity, well-being, and quality of life.

## Data Availability

The data are available at: https://osf.io/gcxev.
