# Supplementary material for: Hope in working-age adults with irreversible vision loss: a scoping review
Source: Rev Esc Enferm USP. 2026 Aug 14;60:e20260181. doi: 10.1590/1980-220X-REEUSP-2026-0181en (PMC13489688; doi:10.1590/1980-220X-REEUSP-2026-0181en)
Supplement: Translation

# Translation: Esperança em adultos em idade ativa com perda irreversível da visão: *scoping review*

**DOI:** 10.1590/1980-220X-REEUSP-2026-0181pt

**Authors:** Cátia Isabel Marranita dos Santos Duarte, Ana Isabel Batalha Bicho, Mónica Figueiredo da Silva Bento, Amélia Simões Figueiredo, Ana Resende

**Affiliations:** 1 Universidade Católica Portuguesa, Faculdade de Ciências da Saúde e Enfermagem, Lisboa, Portugal.; 2 Unidade Local de Saúde de Almada Seixal, Almada, Setúbal, Portugal.

**Keywords:** Adulto, Cegueira, Enfermagem, Esperança, Habilidades de Enfrentamento

## Abstract

**Objetivo:**

Mapear a evidência científica existente sobre a esperança em adultos em idade ativa face à perda irreversível da visão, identificando conceitos, abordagens e lacunas na literatura.

**Método:**

Foi realizada uma *scoping review*, de acordo com a metodologia do JBI e orientações do PRISMA-ScR, em bases de dados internacionais e literatura cinzenta, abrangendo estudos publicados entre 2000 e 2025. Os estudos identificados foram submetidos a um processo de levantamento, seleção, extração e síntese temática dos dados por dois revisores independentes, com apoio do *software* NVivo®.

**Resultados:**

Dos 261 estudos identificados na pesquisa bibliográfica, seis cumpriram os critérios de inclusão. Da análise dos estudos, foi possível identificar quatro temas principais: 1 – Conceito e fontes de esperança; 2 – Esperança enquanto mecanismo de *coping*; 3 – Ajustamento psicossocial; 4 – Implicações para a prática clínica. A esperança surge como um fenômeno multidimensional e dinâmico, com impacto direto na adaptação, na qualidade de vida e no significado atribuído à experiência de perda visual.

**Conclusão:**

A esperança desempenha um papel estruturante no processo de adaptação à perda de visão em pessoas em idade ativa, com implicações relevantes para o desenvolvimento de práticas de enfermagem centradas na pessoa. O aprofundamento da investigação nessa área contribuirá para consolidar o conhecimento teórico da ciência da enfermagem e para a orientação de intervenções clínicas promotoras da esperança.

## INTRODUÇÃO

A perda irreversível da visão constitui um evento simultaneamente traumático e profundamente desafiante, particularmente quando ocorre em indivíduos em idade ativa, fase em que a identidade pessoal e social está fortemente ancorada na autonomia, na produtividade e no exercício de papéis familiares, profissionais e sociais. Quando vivida nesse período do ciclo vital, a perda visual configura uma transição complexa em termos de saúde, desencadeando repercussões profundas na identidade, nos projetos de vida e nos modos de relação da pessoa consigo própria, com os outros e com o mundo.

A faixa etária entre 18 e 65 anos é delimitada por corresponder ao período habitualmente associado à idade ativa, no qual a identidade adulta está fortemente relacionada à autonomia, à participação sociolaboral e ao desempenho de papéis familiares, profissionais e sociais. Nessa fase do ciclo vital, o trabalho e os papéis sociais assumem uma relevância particular na construção do *self*, no sentido de competência e no bem-estar psicológico^(1,2)^. Assim, a perda irreversível da visão pode representar uma rutura significativa nos projetos de vida, na participação social e na integração profissional, tornando pertinente analisar a esperança enquanto recurso adaptativo no processo de transição vivido por essa população^(1,2,3)^.

A visão desempenha um papel estruturante na construção do *self* e na interação com o contexto, pois a sua perda implica uma reorganização significativa do sentido de continuidade, das expectativas face ao futuro e das referências que sustentam a vida cotidiana. Evidência recente demonstra que a perda visual afeta de forma direta a qualidade de vida, os planos para o futuro e o bem-estar psicológico, podendo representar um elevado ónus emocional e psicossocial para a pessoa afetada, exigindo processos adaptativos complexos que vão muito além da limitação funcional^(4)^.

A perda visual surge também associada a um forte impacto psicológico, desencadeando um conjunto de respostas emocionais e comportamentais que exigem ajustamento adaptativo contínuo, com implicações na saúde mental e no bem-estar psicológico da pessoa afetada^(4)^.

Estudos qualitativos apontam que a perda de visão severa afeta profundamente a vivência cotidiana, a participação social e a percepção do sentido da vida, indicando que a adaptação envolve múltiplos domínios da experiência humana^(5)^.

Revisões sobre bem-estar em adultos com deficiência visual destacam que a adaptação psicossocial depende de fatores interligados, incluindo identidade pessoal, suporte social e sentido de coerência de vida^(6)^.

Nesse contexto, a esperança surge não apenas como um estado emocional, mas como um processo dinâmico de adaptação, permitindo à pessoa reconstruir significado, redefinir objetivos e encontrar novas formas de participação e propósito. Apesar de a sua relevância reconhecida em contextos de doença crônica e transição em saúde, a evidência relativa à esperança em pessoas em idade ativa com perda irreversível da visão permanece dispersa e pouco sistematizada^(7,8)^.

Embora a literatura em oftalmologia tenha vindo a valorizar progressivamente as dimensões subjetivas e psicossociais da perda visual^(9,10)^, a compreensão da esperança enquanto fenômeno associado à experiência vivida e aos processos de adaptação permanece ainda pouco sistematizada^(3,8,10)^. Nesse sentido, torna-se pertinente aprofundar o conhecimento sobre a forma como a esperança se manifesta em pessoas em idade ativa com perda irreversível da visão, particularmente no contexto da transição, do ajustamento psicossocial e da reconstrução de significado face à nova condição de vida.

Assim, esta *scoping review* visa mapear a literatura existente sobre esse fenômeno, contribuindo para fundamentar práticas de cuidado mais humanizadas e centradas na pessoa.

## MÉTODO

### Desenho do Estudo

A pesquisa foi realizada em novembro de 2025, de acordo com o manual do JBI e as orientações do *Preferred Reporting Items for Systematic reviews and Meta-Analyses extension for Scoping Reviews* (PRISMA-ScR). A estratégia completa da pesquisa, incluindo as combinações de termos utilizadas em cada base de dados, está disponível para consulta no protocolo previamente registado no *Open Science Framework* ^(11)^, com o DOI 10.17605/OSF.IO/GCXEV, de acordo com os princípios de transparência e replicabilidade. A questão de investigação que norteou esta revisão foi: de que forma a esperança influencia o processo de transição e as estratégias de *coping* de pessoas em idade ativa que enfrentam a perda irreversível da visão? O desenho metodológico foi estruturado segundo o PCC (População, Conceito e Contexto)^(11)^.

### População e Critérios de Seleção

A população incluiu adultos em idade ativa/laboral, entre 18 e 65 anos, com perda irreversível da visão, independentemente da etiologia. O conceito central analisado foi a esperança enquanto fenômeno psicológico, social e espiritual associado ao processo de adaptação e transição. O contexto incluiu todos os cenários, abrangendo perspectivas clínicas, sociais e comunitárias.

Foram incluídos estudos publicados entre 2000 e 2025 em língua portuguesa, inglesa ou espanhola. Foram excluídos estudos que abordassem condições de perda visual reversível ou diagnóstico de cegueira ocorrido antes dos 18 anos ou após 65 anos.

### Coleta de Dados

A pesquisa bibliográfica foi realizada nas bases de dados MEDLINE *Complete*, Scopus e *Web of Science*, bem como na literatura cinzenta através da *ProQuest Dissertations & Theses Citation Index*. As estratégias de pesquisa combinaram termos livres e descritores *Medical Subject Headings* relacionados à esperança, à perda de visão e à idade ativa, adaptadas a cada base de dados.

Os registos identificados foram importados para a plataforma Rayyan, onde os duplicados foram removidos e a triagem dos títulos e resumos foi realizada por dois revisores independentes. Os artigos potencialmente elegíveis foram posteriormente avaliados na íntegra, mantendo-se o processo de revisão em duplicado, com recurso a um terceiro revisor em caso de divergência.

### Análise e Tratamento de Dados

Os dados foram analisados de forma descritiva e temática, com apoio do *software* NVivo® (Lumivero, versão 14), com os resultados sendo organizados em temas e subtemas.

A análise de dados seguiu uma abordagem temática de natureza predominantemente indutiva, permitindo a identificação de padrões, temas e significados emergentes da evidência incluída. O processo analítico envolveu a leitura aprofundada dos estudos, a codificação dos excertos relevantes e o posterior agrupamento em categorias temáticas e subtemáticas relacionadas à esperança, às estratégias de *coping*, ao ajustamento psicossocial e às implicações para a prática clínica.

O *software* NVivo*®* (Lumivero, versão 14) foi utilizado como ferramenta de apoio à organização, à gestão e à codificação dos dados qualitativos extraídos dos estudos, facilitando a sistematização dos conteúdos e a identificação de relações entre temas emergentes.

Foram extraídas variáveis relativas às características metodológicas dos estudos (autor, ano, país, método de estudo, população, contexto e objetivos), bem como dados relacionados à conceptualização da esperança, aos fatores promotores e inibidores, aos mecanismos de adaptação, ao impacto psicossocial da perda visual e às implicações para a prática de enfermagem.

### Aspectos Éticos

Foram respeitados os princípios éticos da investigação científica, nomeadamente no que se refere à integridade, ao rigor metodológico, à transparência do processo de revisão e à correta referenciação das fontes utilizadas, em conformidade com as orientações internacionais para revisões de literatura.

## RESULTADOS

A pesquisa bibliográfica resultou na identificação de 261 registos. Após a remoção de duplicados e a aplicação dos critérios de inclusão e exclusão, seis estudos foram incluídos nesta *scoping review*. O processo de identificação, triagem, elegibilidade e inclusão encontra-se ilustrado no diagrama de fluxo do PRISMA-ScR (Figura 1).

**Figura 1:** Diagrama de fluxo do Preferred Reporting Items for Systematic reviews and Meta-Analyses extension for Scoping Reviews

As publicações incluídas se distribuem entre 2008 e 2022. Dos seis estudos incluídos, dois apresentaram metodologia qualitativa (33,3%), três, quantitativa (50,0%), e um consistiu em um relato/reflexão narrativa (16,7%). Quanto à origem geográfica, verificou-se a predominância de estudos desenvolvidos nos Estados Unidos da América (50,0%) e no Reino Unido (33,3%). Os estudos abordaram, sobretudo, temas como esperança, suporte social, qualidade de vida, experiência vivida e ajustamento psicossocial associados à perda visual irreversível.

Constatou-se a heterogeneidade metodológica, com abordagens quantitativas transversais, qualitativas exploratórias e relatos narrativos, centradas, sobretudo, na experiência subjetiva da perda visual, na esperança, no suporte social, na qualidade de vida e nos processos de adaptação psicossocial.

O Quadro 1 apresenta a síntese das principais características dos estudos incluídos, nomeadamente autores, ano de publicação, país de origem, método, população, objetivos e principais resultados.

**Quadro 1:** Síntese das principais características dos estudos incluídos – Lisboa, Portugal, 2026.

Título do artigo	Autores/ano	País de origem	Método	População	Objetivo	Principais resultados
*Disability Self-Worth and Positive Personal Meaning in Disability: Correlates of Hope Among U.S. Residents With Physical Disabilities* ^(7)^	Zapata MA (2022)	EUA	Quantitativo transversal	Adultos com deficiência física e sensorial	Analisar a relação entre identidade da deficiência, significado pessoal, autoestima e esperança.	O sentido de valor pessoal e o significado positivo atribuído à deficiência se associaram significativamente a níveis mais elevados de esperança.
*Hope and Social Support in Adults Who Are Legally Blind at a Training Center* ^(8)^	Singletary C, Goodwyn MA, Carter AP (2009)	EUA	Quantitativo correlacional transversal	Adultos legalmente cegos em centro de reabilitação	Examinar a relação entre esperança e suporte social.	A esperança apresentou associação positiva ao suporte social percebido, incluindo o apoio de amigos, família e pessoa significativa.
*Impact of Low Vision on Well-Being in 10 European Countries* ^(12)^	Mojon-Azzi SM, Sousa-Poza A, Mojon DS (2008)	Dez países europeus	Quantitativo observacional	Pessoas com idade ≥50 anos	Analisar a associação entre baixa visão e bem-estar.	A baixa visão associou-se à pior bem-estar emocional, à menor esperança no futuro, à tristeza, à fadiga, à irritabilidade e a dificuldades de concentração.
*Information Gives Hope* ^(10)^	Lesselroth M (2019)	EUA	Relato narrativo/ reflexivo	Pessoa adulta com glaucoma	Descrever a experiência face ao diagnóstico e ao papel da informação.	A informação clara, recursos acessíveis e o suporte de pares favoreceram a esperança, o controlo e a adaptação ao diagnóstico.
*Seeing Beyond Anatomy: Quality of Life with Geographic Atrophy* ^(9)^	Caswell D, Caswell W, Carlton J (2021)	Canadá/Reino Unido	Qualitativo/ comentário com perspectiva de doente e cuidador	Pessoa com atrofia geográfica e cuidador informal	Explorar o impacto da atrofia geográfica na qualidade de vida.	A perda visual afetou a autonomia, os *hobbies*, as relações sociais e a qualidade de vida. Nesse contexto, a informação, o suporte e as tecnologias assistivas emergiram como recursos relevantes.
*Experiencing Motherhood as a Blind Mother in the Greater Accra Region of Ghana; a Qualitative Study* ^(3)^	Acheampong AK, Marfo M, Aziato L (2022)	Gana	Qualitativo exploratório-descritivo	19 mães cegas	Explorar a experiência de maternidade em mulheres cegas.	Emergiram dificuldades no papel materno, discriminação, sofrimento psicológico e estratégias de *coping* centradas na oração, na automotivação e na esperança nos filhos.

Legenda: EUA – Estados Unidos da América.

A análise temática permitiu identificar quatro temas principais: (1) Conceito e fontes de esperança; (2) Esperança enquanto mecanismo de *coping*; (3) Ajustamento psicossocial; e (4) Implicações e lacunas para a prática.

O Quadro 2 apresenta a síntese dos resultados, organizados de acordo com os temas identificados, incluindo excertos representativos e os respetivos estudos de origem.

**Quadro 2:** Síntese dos resultados, organizados de acordo com os temas identificados – Lisboa, Portugal, 2026.

Temas principais	Tema e subtemas	Excerto literal (en)
1. Conceito e fontes de esperança	1 – Conceito e fontes de esperança	“*Hope is a process of thinking about one’s goals...*”^(7)^ “*Cognitive motivational system involving both goal-directed motivation and consideration of means to achieve goals.*”^(7)^ “*Hope has been associated with various positive psychological, physical health, and academic outcomes.*”^(7)^ “*Self-worth and personal meaning predicted higher hope.*”^(7)^ *“Hope is a process of thinking about one’s goals that involves: (a) the motivation to move toward those goals (agency); and (b) the consideration of means to achieve those goals*.”^(7)^ “*Knowing about the current evidence... helps patients stay positive.*”^(10)^
	1.1 – Fatores promotores de esperança	“*High levels of hope were tied to strong support networks*.”^(8)^ *“The length of time spent at training centers would be associated with levels of self-esteem and hope*.”^(8)^ *“Positive relationships that were found between hope and perceived social support*.”^(8)^ “*Magnifying devices can reduce the degree of handicap.*” ^(8)^ “*Prayer was done in such a way that, they could talk to God at different times.*”^(3)^ “*It would have helped to have a list of resources to take home after the diagnosis.*”^(10)^ *“Having access to information* (...) *and knowing what developments are being worked on gives me hope and peace of mind.*”^(10)^
	1.2 – Fatores obstrutores de esperança	“*Patients lack information around their expected prognosis...*”^(9)^
2 – Esperança e processo de adaptação/*coping*	2.1 *– Coping* ativo ou proativo	“*I took matters into my own hands... started my own support group.*”^(10)^
	2.2 *– Coping* centrado na emoção ou espiritual	“*Prayer was the safe haven...*”^(3)^ “*Participants* (...) *coped with their challenges with prayer, hope in their children and self-motivation.*”^(3)^ *“I hope I’m at the plateau of visual loss, but I don’t know*.”^(9)^
	2.3 – Relação com a identidade pessoal	“*Hope has been positively associated with self-esteem...*”^(7)^
	2.4 – Resiliência e autoeficácia	“*Developing strength in adversity...*”^(7)^ “*The long-term blindness of these individuals may have bolstered their self-acceptance and adjustment to blindness.*”^(8)^
3 – Ajustamento psicossocial	3.1 – Suporte social e interpessoal	“*Hope was tied to strong social support networks...*”^(8)^ “*Their children were their source of hope...*”^(3)^
	3.2 – Qualidade de vida e bem-estar	“*Persons with low vision... more likely to wish for death.*”^(9)^
	3.3 – *Outcomes* psicológicos	“*Identifying and encouraging development of strengths...*”^(7)^
4 – Implicações para a prática e lacunas no conhecimento	4.1 – Sugestões de intervenções para a prática	“*Let patients know there is hope... make them aware of resources*.”^(10)^ “*Make patients aware of the resources available to them, including online support groups.*”^(10)^ *“Knowing about the current evidence on disease progression and sharing it with patients can help them stay positive.”* ^(10)^
	4.2 – Lacunas da literatura	“*Research has yet to explore disability self-worth...*”(7)

De forma transversal aos estudos analisados, a esperança emerge como um recurso multidimensional, integrando componentes cognitivas, relacionais e existenciais. A análise temática evidencia que o acesso à informação, o suporte social significativo e a reconstrução do significado pessoal funcionam como fatores promotores convergentes da esperança, influenciando positivamente o ajustamento psicossocial e as estratégias de *coping* face à perda irreversível da visão^(3,8,9,10)^, tal como é apresentado na Figura 2.

**Figura 2:** Síntese dos achados.

## DISCUSSÃO

A abordagem interpretativa adotada neste estudo é orientada pela compreensão da esperança enquanto fenômeno complexo no contexto dos processos de saúde, integrando a experiência da pessoa, os processos de transição e os contextos de cuidado. Essa perspectiva permite analisar, de forma articulada, as dimensões individuais e relacionais que influenciam a esperança, contribuindo para a compreensão das respostas humanas e para o desenvolvimento de intervenções de enfermagem mais ajustadas à prática clínica. Ao evidenciar a esperança como um recurso adaptativo relacional e situado, os resultados desta *scoping review* reforçam a pertinência de leituras que ultrapassam uma perspectiva biomédica da perda visual, valorizando dimensões subjetivas, sociais e existenciais que influenciam o ajustamento biopsicossocial.

À luz dos estudos incluídos, a esperança emerge como um constructo complexo, predominantemente compreendido como um processo cognitivo-motivacional orientado para objetivos, mais do que como um estado emocional isolado^(3,7,8,12)^.

A evidência analisada sustenta a esperança como a capacidade de mobilizar motivação para a prossecução de objetivos significativos e, simultaneamente, de identificar possibilidades concretas para alcançá-los, mesmo em contextos de adversidade^(7,8,12)^.

Essa conceptualização é corroborada por resultados empíricos que demonstram associações consistentes entre níveis mais elevados de esperança e indicadores positivos de ajustamento psicológico, nomeadamente autoestima, sentido de valor pessoal e significado atribuído à experiência vivida, reforçando o papel da esperança como recurso adaptativo central^(3,7,8)^.

No conjunto dos estudos analisados, a esperança assume uma expressão fortemente relacional e contextual, sendo influenciada por fatores que podem simultaneamente favorecê-la ou fragilizá-la^(3,8,9,10,13)^.

O acesso a informações claras, compreensíveis e atempadas sobre o diagnóstico, a progressão da condição e os recursos disponíveis emergem de forma consistente como um fator facilitador da esperança, pois permite antecipar mudanças, gerir expectativas e reduzir a incerteza associada ao futuro^(3,8,9,13)^.

De igual modo, a presença de redes de suporte social significativas, incluindo pares, profissionais e contextos formativos ou de reabilitação, associa-se a níveis mais elevados de esperança, funcionando como facilitadora dos mecanismos de *coping* e do ajustamento psicossocial^(3,8,10,13)^.

Em contraste, contextos marcados por desinformação, ausência de acompanhamento, estigmatização ou atitudes sociais negativas surgem associados à fragilização da esperança, acentuando sentimentos de incerteza, isolamento e perda de controlo^(3,10,13)^.

No contexto específico da perda irreversível da visão em idade ativa, a esperança emerge como um fenômeno central no processo de adaptação, configurando-se como um recurso adaptativo relacional e processual^(7,8,9,10,12)^.

Nesse enquadramento, a esperança está relacionada à autoestima e ao sentido de valor pessoal, à percepção de capacidade para reorganizar objetivos e rotinas, à construção de significado diante da perda visual (incluindo a reconstrução identitária e a reconfiguração de papéis socialmente valorizados) e do suporte social percebido^(7,8,10,12)^.

Assim, a esperança não se manifesta como a negação da irreversibilidade da condição, mas como a capacidade de reorganizar projetos de vida, encontrar soluções possíveis e atribuir continuidade e sentido à experiência, mesmo perante limitações funcionais progressivas, refletindo a natureza multidimensional, dinâmica e situada do ajustamento psicossocial nesse contexto^(3,8,9,10)^.

A análise da qualidade de vida em contextos de perda visual irreversível sugere que os processos de adaptação vão além da dimensão anatômica ou funcional, emergindo principalmente da forma como as pessoas reinterpretam sua experiência vivida, reconstroem sua identidade e redefinem o que significa viver bem diante da progressão da condição. Nessa perspectiva, a esperança manifesta-se de modo implícito como prospecto de continuidade, de propósito e de valor atribuído à vida, mesmo face a limitações funcionais progressivas e irreversíveis^(3,9)^.

Os estudos analisados evidenciam que a informação fornecida aos doentes desempenha um papel estruturante na construção e manutenção da esperança, não se limitando a uma função instrumental, mas assumindo-se como um elemento central na promoção do senso de controle, previsibilidade e capacidade de antecipação do futuro. O acesso a informações claras, compreensíveis e ajustadas à pessoa, particularmente no que se refere ao prognóstico, à progressão da perda visual e aos recursos terapêuticos, de reabilitação e sociais disponíveis, emerge consistentemente como um fator que facilita o ajustamento psicológico. Isso contribui para a redução da incerteza, do medo e de sentimentos de desesperança, além de sustentar processos mais adaptativos de *coping* e reorganização da vida cotidiana^(3,7,8,9,10)^.

Apesar da relevância do fenômeno, a literatura revela lacunas significativas, nomeadamente no desenvolvimento e na avaliação de intervenções estruturadas promotoras de esperança, particularmente no âmbito da enfermagem. Nesse sentido, o cuidado em enfermagem integrado na comunicação terapêutica permite trabalhar a esperança através da educação para a saúde e o acompanhamento continuado, transformando a informação em conhecimento significativo, promovendo a participação ativa da pessoa e sustentando processos de adaptação mais autónomos, dignos e psicossocialmente ajustados diante da perda irreversível da visão em idade ativa.

### Limitações do Estudo

Apesar do rigor metodológico adotado, esta *scoping review* apresenta algumas limitações que importa reconhecer. O número reduzido de referências incluídas nesta revisão é reflexo da especificidade do fenômeno investigado e da escassez de investigação publicada que aborda de forma direta e sistemática a esperança no contexto da perda irreversível da visão em idade ativa, motivo pelo qual se optou por alargar o período temporal aos últimos 25 anos. Além da escassez, os estudos disponíveis foram desenvolvidos em contextos heterogêneos quanto à idade, à etiologia da deficiência visual e ao enquadramento sociocultural.

A predominância de abordagens qualitativas e exploratórias, frequentemente assentes em amostras reduzidas, limita a transferibilidade dos resultados. Adicionalmente, a diversidade conceptual na definição de esperança, ora descrita como recurso cognitivo-motivacional, ora como experiência existencial ou espiritual, dificulta a comparação entre estudos e a consolidação de um enquadramento teórico unificado.

Verifica-se, ainda, uma escassa exploração da dimensão temporal da esperança, sendo raros os estudos que analisam a sua evolução ao longo do processo de adaptação à perda visual. Por fim, a ausência de estudos de intervenção limita a extrapolação dos achados para a prática clínica baseada na evidência.

### Sugestões de Melhoria e Investigação Futura

Os resultados desta *scoping review* reforçam a pertinência e relevância do desenvolvimento de investigação aprofundada sobre o processo de transição vivido por pessoas adultas em idade ativa face à perda de visão. A articulação entre esperança, significado atribuído à experiência, estratégias de *coping*, reconstrução identitária e suporte relacional, identificada na literatura analisada, aponta para a necessidade de se compreender esse fenômeno de maneira processual e contextualizada, integrando as dimensões biológica, psicológica, social e espiritual da experiência vivida. Esses achados sustentam a opção por uma abordagem qualitativa de natureza interpretativa, como uma Teoria Fundamentada nos Dados Construtivista, enquanto uma metodologia capaz de captar a complexidade do processo de transição e de dar voz às pessoas na construção dos significados associados à perda visual.

Estudos futuros deverão também privilegiar desenhos longitudinais, que permitam compreender a evolução da esperança desde o diagnóstico até fases mais avançadas de adaptação e reconstrução identitária, aprofundando a investigação sobre a esperança como processo dinâmico no contexto da perda irreversível da visão.

Recomenda-se também a validação de instrumentos sensíveis à experiência subjetiva da esperança em pessoas com deficiência visual como outra lacuna relevante.

No domínio da prática clínica, são necessários estudos de intervenção que avaliem estratégias estruturadas de promoção da esperança, por meio de programas educacionais, acompanhamento de enfermagem focado na transição e facilitação do acesso à informação e a redes de apoio, visando proporcionar cuidados mais humanizados e centrados no paciente^(3,7,8,9,10,11,12)^.

## CONCLUSÃO

Esta *scoping review* evidencia, de forma consistente, a esperança como um recurso adaptativo central no processo de transição vivido por pessoas em idade ativa face à perda irreversível da visão. Os resultados demonstram que a esperança se configura como um fenômeno dinâmico, relacional e contextual, estreitamente articulado com a autoestima, o significado pessoal, o suporte social e o acesso a informações claras e relevantes, desempenhando um papel determinante no ajustamento psicossocial, na reorganização dos projetos de vida e na reconstrução identitária.

Apesar desses contributos, a análise da literatura revela uma produção científica ainda limitada e fragmentada, caracterizada pela predominância de abordagens exploratórias, pela escassez de estudos longitudinais e pela inexistência de intervenções estruturadas orientadas para a promoção da esperança, particularmente no âmbito da enfermagem, o que reforça a necessidade de aprofundar a investigação e de desenvolver modelos conceptuais integradores que sustentem práticas clínicas baseadas na evidência.

Nesse contexto, a enfermagem emerge como ciência na mediação da esperança, através de práticas centradas na pessoa que valorizem a comunicação terapêutica, a educação para a saúde, o acompanhamento ao longo da transição e a facilitação do acesso a recursos e redes de apoio, contribuindo para cuidados mais humanizados, integrados e sensíveis à experiência vivida, com impacto na dignidade, no bem-estar e na qualidade de vida dessas pessoas.

## Data Availability

Os dados estão disponíveis em: https://osf.io/gcxev.

# Translation: Esperanza en adultos en edad laboral con pérdida irreversible de la visión: una revisión de alcance

**DOI:** 10.1590/1980-220X-REEUSP-2025-0464es

**Authors:** Cátia Isabel Marranita dos Santos Duarte, Ana Isabel Batalha Bicho, Mónica Figueiredo da Silva Bento, Amélia Simões Figueiredo, Ana Resende

**Affiliations:** 1 Universidade Católica Portuguesa, Faculdade de Ciências da Saúde e Enfermagem, Lisboa, Portugal.; 2 Unidade Local de Saúde de Almada Seixal, Almada, Setúbal, Portugal.

**Keywords:** Adulto, Ceguera, Enfermería, Esperanza, Habilidades de Afrontamiento

## Abstract

**Objetivo:**

Mapear la evidencia científica existente sobre la esperanza en adultos en edad laboral ante la pérdida irreversible de la visión, identificando conceptos, enfoques y lagunas en la literatura.

**Método:**

Se llevó a cabo una revisión de alcance (scoping review), de acuerdo con la metodología del JBI y las directrices de PRISMA-ScR, en bases de datos internacionales y literatura gris, abarcando estudios publicados entre 2000 y 2025. Los estudios identificados fueron sometidos a un proceso de recopilación, selección, extracción y síntesis temática de los datos por parte de dos revisores independientes, con el apoyo del *software* NVivo®.

**Resultados:**

De los 261 estudios identificados en la búsqueda bibliográfica, seis cumplieron con los criterios de inclusión. A partir del análisis de los estudios, se pudieron identificar cuatro temas principales: 1 – Concepto y fuentes de esperanza; 2 – La esperanza como mecanismo de *coping*; 3 – Adaptación psicosocial; 4 – Implicaciones para la práctica clínica. La esperanza se presenta como un fenómeno multidimensional y dinámico, con un impacto directo en la adaptación, la calidad de vida y el significado que se le atribuye a la experiencia de la pérdida de la visión.

**Conclusión:**

La esperanza desempeña un papel fundamental en el proceso de adaptación a la pérdida de la visión en personas en edad activa, con implicaciones relevantes para el desarrollo de prácticas de enfermería centradas en la persona. La profundización de la investigación en esta área contribuirá a consolidar el conocimiento teórico de la ciencia de la enfermería y a orientar las intervenciones clínicas que promuevan la esperanza.

## INTRODUCCIÓN

La pérdida irreversible de la visión constituye un evento a la vez traumático y profundamente desafiante, particularmente cuando ocurre en personas en edad activa, etapa en la que la identidad personal y social está fuertemente anclada en la autonomía, la productividad y el ejercicio de roles familiares, profesionales y sociales. Cuando se vive en este período del ciclo vital, la pérdida de la visión supone una transición compleja en términos de salud, lo que desencadena profundas repercusiones en la identidad, en los proyectos de vida y en las formas de relación de la persona consigo misma, con los demás y con el mundo.

El rango de edad entre los 18 y los 65 años se delimita por corresponder al período habitualmente asociado a la edad activa, en el que la identidad adulta está fuertemente relacionada con la autonomía, la participación sociolaboral y el desempeño de roles familiares, profesionales y sociales. En esta etapa del ciclo vital, el trabajo y los roles sociales adquieren una relevancia especial en la construcción del *yo*, en el sentido de la competencia y el bienestar psicológico^(1,2)^. Por lo tanto, la pérdida irreversible de la visión puede representar una ruptura significativa en los proyectos de vida, la participación social y la integración profesional, lo que hace pertinente analizar la esperanza como recurso adaptativo en el proceso de transición que vive esta población^(1,2,3)^.

La visión desempeña un papel fundamental en la construcción del *yo* y en la interacción con el contexto, ya que su pérdida implica una reorganización significativa del sentido de continuidad, de las expectativas respecto al futuro y de las referencias que sustentan la vida cotidiana. Evidencia reciente demuestra que la pérdida de la visión afecta directamente la calidad de vida, los planes para el futuro y el bienestar psicológico, y puede representar una carga emocional y psicosocial elevada para la persona afectada, lo que exige procesos adaptativos complejos que van mucho más allá de la limitación funcional^(4)^.

La pérdida de la visión también se asocia con un fuerte impacto psicológico, lo que desencadena un conjunto de respuestas emocionales y conductuales que exigen un ajuste adaptativo continuo, con implicaciones en la salud mental y el bienestar psicológico de la persona afectada^(4)^.

Los estudios cualitativos señalan que la pérdida grave de la visión afecta profundamente la vida cotidiana, la participación social y la percepción del sentido de la vida, lo que indica que la adaptación involucra múltiples ámbitos de la experiencia humana^(5)^.

Las revisiones sobre el bienestar en adultos con discapacidad visual destacan que la adaptación psicosocial depende de factores interrelacionados, entre ellos la identidad personal, el apoyo social y el sentido de coherencia de la vida^(6)^.

En este contexto, la esperanza surge no solo como un estado emocional, sino como un proceso dinámico de adaptación, que permite a la persona reconstruir el significado, redefinir objetivos y encontrar nuevas formas de participación y propósito. A pesar de que se reconoce su relevancia en contextos de enfermedad crónica y transición en salud, la evidencia relativa a la esperanza en personas en edad activa con pérdida irreversible de la visión sigue siendo dispersa y poco sistematizada^(7,8)^.

Aunque la literatura en oftalmología ha ido valorando progresivamente las dimensiones subjetivas y psicosociales de la pérdida de la visión^(9,10)^, la comprensión de la esperanza como fenómeno asociado a la experiencia vivida y a los procesos de adaptación sigue siendo poco sistematizada^(3,8,10)^. En este sentido, resulta pertinente profundizar en el conocimiento sobre la forma en que se manifiesta la esperanza en personas en edad activa con pérdida irreversible de la visión, particularmente en el contexto de la transición, la adaptación psicosocial y la reconstrucción de significado ante la nueva condición de vida.

Así, esta revisión de alcance tiene como objetivo mapear la literatura existente sobre este fenómeno, contribuyendo a fundamentar prácticas de atención más humanizadas y centradas en la persona.

## MÉTODO

### Diseño del Estudio

La investigación se llevó a cabo en noviembre de 2025, de acuerdo con el manual del JBI y las directrices de los *Preferred Reporting Items for Systematic reviews and Meta-Analyses extension for Scoping Reviews* (PRISMA-ScR). La estrategia completa de la investigación, incluidas las combinaciones de términos utilizadas en cada base de datos, está disponible para su consulta en el protocolo previamente registrado en el *Open Science Framework* ^(11)^, con el DOI 10.17605/OSF.IO/GCXEV, de acuerdo con los principios de transparencia y replicabilidad. La pregunta de investigación que guió esta revisión fue: ¿de qué manera influye la esperanza en el proceso de transición y en las estrategias de *coping* de las personas en edad activa que enfrentan la pérdida irreversible de la visión? El diseño metodológico se estructuró según el PCC (Población, Concepto y Contexto)^(11)^.

### Población y Criterios de Selección

La población incluyó a adultos en edad activa o laboral, de entre 18 y 65 años, con pérdida irreversible de la visión, independientemente de la etiología. El concepto central analizado fue la esperanza como fenómeno psicológico, social y espiritual asociado al proceso de adaptación y transición. El contexto incluyó todos los escenarios, abarcando perspectivas clínicas, sociales y comunitarias.

Se incluyeron estudios publicados entre 2000 y 2025 en portugués, inglés o español. Se excluyeron los estudios que abordaran condiciones de pérdida visual reversible o un diagnóstico de ceguera ocurrido antes de los 18 años o después de los 65 años.

### Recolección de Datos

La búsqueda bibliográfica se realizó en las bases de datos MEDLINE *Complete*, Scopus y *Web of Science*, así como en la literatura gris a través del *ProQuest Dissertations & Theses Citation Index*. Las estrategias de búsqueda combinaron términos libres y descriptores *Medical Subject Headings* relacionados con la esperanza, la pérdida de la visión y la edad activa, adaptadas a cada base de datos.

Los registros identificados se importaron a la plataforma Rayyan, donde se eliminaron los duplicados y dos revisores independientes se encargaron de la selección de los títulos y resúmenes. Posteriormente, se evaluaron en su totalidad los artículos potencialmente elegibles, manteniendo el proceso de revisión por pares, con el apoyo de un tercer revisor en caso de discrepancia.

### Análisis y Procesamiento de Datos

Los datos se analizaron de manera descriptiva y temática, con el apoyo del *software* NVivo^®^ (Lumivero, versión 14), y los resultados se organizaron en temas y subtemas.

El análisis de datos siguió un enfoque temático de naturaleza predominantemente inductiva, lo que permitió identificar patrones, temas y significados emergentes a partir de la evidencia incluida. El proceso analítico implicó la lectura exhaustiva de los estudios, la codificación de los fragmentos relevantes y su posterior agrupación en categorías temáticas y subtemáticas relacionadas con la esperanza, las estrategias de *coping*, la adaptación psicosocial y las implicaciones para la práctica clínica.

Se utilizó el *software* NVivo^*®*^ (Lumivero, versión 14) como herramienta de apoyo para la organización, la gestión y la codificación de los datos cualitativos extraídos de los estudios, lo que facilitó la sistematización de los contenidos y la identificación de relaciones entre los temas emergentes.

Se extrajeron variables relacionadas con las características metodológicas de los estudios (autor, año, país, método de estudio, población, contexto y objetivos), así como datos relacionados con la conceptualización de la esperanza, los factores promotores e inhibidores, los mecanismos de adaptación, el impacto psicosocial de la pérdida de la visión y las implicaciones para la práctica de la enfermería.

### Aspectos Éticos

Se respetaron los principios éticos de la investigación científica, en particular en lo que se refiere a la integridad, el rigor metodológico, la transparencia del proceso de revisión y la correcta citación de las fuentes utilizadas, de conformidad con las directrices internacionales para las revisiones bibliográficas.

## RESULTADOS

La búsqueda bibliográfica permitió identificar 261 registros. Tras eliminar los duplicados y aplicar los criterios de inclusión y exclusión, se incluyeron seis estudios en esta revisión de alcance. El proceso de identificación, selección, elegibilidad e inclusión se ilustra en el diagrama de flujo de PRISMA-ScR (Figura 1).

**Figura 1:** Diagrama de flujo de la Extensión de los Elementos de Reportaje Preferidos para Revisiones S

Las publicaciones incluidas abarcan el período comprendido entre 2008 y 2022. De los seis estudios incluidos, dos presentaron una metodología cualitativa (33,3 %), tres, cuantitativa (50,0 %), y uno consistió en un relato o reflexión narrativa (16,7 %). En cuanto al origen geográfico, se observó un predominio de estudios realizados en los Estados Unidos de América (50,0 %) y en el Reino Unido (33,3 %). Los estudios abordaron, sobre todo, temas como la esperanza, el apoyo social, la calidad de vida, la experiencia vivida y la adaptación psicosocial asociados a la pérdida visual irreversible.

Se observó una heterogeneidad metodológica, con enfoques cuantitativos transversales, cualitativos exploratorios y relatos narrativos, centrados principalmente en la experiencia subjetiva de la pérdida de la visión, la esperanza, el apoyo social, la calidad de vida y los procesos de adaptación psicosocial.

El Cuadro 1 presenta un resumen de las principales características de los estudios incluidos, a saber: autores, año de publicación, país de origen, método, población, objetivos y resultados principales.

**Cuadro 1:** Resumen de las principales características de los estudios incluidos – Lisboa, Portugal, 2026.

Título del artículo	Autores/año	País de origen	Método	Población	Objetivo	Principales resultados
*Disability Self-Worth and Positive Personal Meaning in Disability: Correlates of Hope Among U.S. Residents With Physical Disabilities* ^(7)^	Zapata MA (2022)	EUA	Cuantitativo transversal	Adultos con discapacidad física y sensorial	Analizar la relación entre la identidad de la discapacidad, el significado personal, la autoestima y la esperanza.	El sentido de valor personal y el significado positivo que se le atribuye a la discapacidad se asociaron de manera significativa con niveles más altos de esperanza.
*Hope and Social Support in Adults Who Are Legally Blind at a Training Center* ^(8)^	Singletary C, Goodwyn MA, Carter AP (2009)	EUA	Estudio cuantitativo correlacional transversal	Adultos con ceguera legal en un centro de rehabilitación	Examinar la relación entre la esperanza y el apoyo social.	La esperanza se asoció positivamente con el apoyo social percibido, incluyendo el apoyo de amigos, familiares y la pareja.
*Impact of Low Vision on Well-Being in 10 European Countries* ^(12)^	Mojon-Azzi SM, Sousa-Poza A, Mojon DS (2008)	Diez países europeos	Estudio cuantitativo observacional	Personas de 50 años o más	Analizar la asociación entre la baja visión y el bienestar.	La baja visión se asoció con un peor bienestar emocional, menor esperanza en el futuro, tristeza, fatiga, irritabilidad y dificultades para concentrarse.
*Information Gives Hope* ^(10)^	Lesselroth M (2019)	EUA	Relato narrativo/ reflexivo	Adultos con glaucoma	Describir la experiencia ante el diagnóstico y el papel de la información.	La información clara, los recursos accesibles y el apoyo de otras personas en situaciones similares favorecieron la esperanza, el control y la adaptación al diagnóstico.
*Seeing Beyond Anatomy: Quality of Life with Geographic Atrophy* ^(9)^	Caswell D, Caswell W, Carlton J (2021)	Canadá/Reino Unido	Cualitativo/ comentario con perspectiva de enfermo y cuidador	Pessoa com atrofia geográfica e cuidador informal	Explorar el impacto de la atrofia geográfica en la calidad de vida.	La pérdida de la visión afectó la autonomía, los *hobbies*, las relaciones sociales y la calidad de vida. En ese contexto, la información, el apoyo y las tecnologías de asistencia se revelaron como recursos relevantes.
*Experiencing Motherhood as a Blind Mother in the Greater Accra Region of Ghana; a Qualitative Study* ^(3)^	Acheampong AK, Marfo M, Aziato L (2022)	Ghana	Cualitativo exploratorio-descriptivo	19 madres ciegas	Explorar la experiencia de la maternidad en mujeres ciegas.	Surgieron dificultades en el rol materno, discriminación, sufrimiento psicológico y estrategias de *coping* centradas en la oración, la automotivación y la esperanza en los hijos.

EUA – Estados Unidos de América.

El análisis temático permitió identificar cuatro temas principales: (1) Concepto y fuentes de esperanza; (2) La esperanza como mecanismo de *coping*; (3) Adaptación psicosocial; y (4) Implicaciones y lagunas para la práctica.

El Cuadro 2 presenta un resumen de los resultados, organizados según los temas identificados, incluyendo extractos representativos y los respectivos estudios de origen.

**Cuadro 2:** Síntesis de los resultados, organizados según los temas identificados – Lisboa, Portugal, 2026.

Temas principales	Tema e subtemas	Extracto literal (inglés)
1. Concepto y fuentes de esperanza	1. Concepto y fuentes de esperanza	“*Hope is a process of thinking about one’s goals...*”^(7)^ “*Cognitive motivational system involving both goal-directed motivation and consideration of means to achieve goals.*”^(7)^ “*Hope has been associated with various positive psychological, physical health, and academic outcomes.*”^(7)^ “*Self-worth and personal meaning predicted higher hope.*”,^(7)^ *“Hope is a process of thinking about one’s goals that involves: (a) the motivation to move toward those goals (agency); and (b) the consideration of means to achieve those goals*.”^(7)^ “*Knowing about the current evidence... helps patients stay positive.*”(10)
	1.1 – Factores promotores de esperanza	“*High levels of hope were tied to strong support networks*.”^(8)^ *“The length of time spent at training centers would be associated with levels of self-esteem and hope*.”^(8)^ *“Positive relationships that were found between hope and perceived social support*.”^(8)^ “*Magnifying devices can reduce the degree of handicap.*” ^(8)^ “*Prayer was done in such a way that, they could talk to God at different times.*”^(3)^ “*It would have helped to have a list of resources to take home after the diagnosis.*”^(10)^ *“Having access to information* (...) *and knowing what developments are being worked on gives me hope and peace of mind.*”^(10)^
	1.2 – Fatores obstructores de esperanza	“*Patients lack information around their expected prognosis...*”^(9)^
2 – Esperanza y proceso de adaptación/*coping*	2.1 *– Coping* activo o proactivo	“*I took matters into my own hands... started my own support group.*”^(10)^
	2.2 *– Coping* centrado en la emoción o espiritual	“*Prayer was the safe haven...*”^(3)^ “*Participants* (...) *coped with their challenges with prayer, hope in their children and self-motivation.*”^(3)^ *“I hope I’m at the plateau of visual loss, but I don’t know*.”^(9)^
	2.3 – Relación con la identidad personal	“*Hope has been positively associated with self-esteem...*”^(7)^
	2.4 – Resiliencia y autoeficácia	“*Developing strength in adversity...*”^(7)^ “*The long-term blindness of these individuals may have bolstered their self-acceptance and adjustment to blindness.*”^(8)^
3 – Adaptación psicosocial	3.1 – Apoyo social e interpersonal	“*Hope was tied to strong social support networks...*”^(8)^ “*Their children were their source of hope...*”^(3)^
	3.2 – Calidad de vida y bienestar	“*Persons with low vision... more likely to wish for death.*”^(9)^
	3.3 – *Outcomes* psicológicos	“*Identifying and encouraging development of strengths...*”^(7)^
4 – Implicaciones para la práctica y brechas de conocimiento	4.1 – Sugestiones de intervenciones para la prática	“*Let patients know there is hope... make them aware of resources*.”^(10)^ “*Make patients aware of the resources available to them, including online support groups.*”^(10)^ *“Knowing about the current evidence on disease progression and sharing it with patients can help them stay positive.”* ^(10)^
	4.2 – Brechas de la literatura	“*Research has yet to explore disability self-worth...*”^(7)^

De manera transversal en los estudios analizados, la esperanza surge como un recurso multidimensional que integra componentes cognitivos, relacionales y existenciales. El análisis temático pone de manifiesto que el acceso a la información, el apoyo social significativo y la reconstrucción del significado personal actúan como factores convergentes que promueven la esperanza, influyendo positivamente en la adaptación psicosocial y en las estrategias de *coping* ante la pérdida irreversible de la visión^(3,8,9,10)^, tal como se presenta en la Figura 2.

**Figura 2:** Resumen de los hallazgos.

## DISCUSIÓN

El enfoque interpretativo adoptado en este estudio se basa en la comprensión de la esperanza como un fenómeno complejo en el contexto de los procesos de salud, integrando la experiencia de la persona, los procesos de transición y los contextos de atención. Esta perspectiva permite analizar, de manera integrada, las dimensiones individuales y relacionales que influyen en la esperanza, lo que contribuye a la comprensión de las respuestas humanas y al desarrollo de intervenciones de enfermería más ajustadas a la práctica clínica. Al poner de manifiesto la esperanza como un recurso adaptativo, relacional y situado, los resultados de esta revisión de alcance refuerzan la pertinencia de interpretaciones que van más allá de una perspectiva biomédica de la pérdida de la visión, valorando las dimensiones subjetivas, sociales y existenciales que influyen en la adaptación biopsicosocial.

A la luz de los estudios incluidos, la esperanza surge como un constructo complejo, entendido predominantemente como un proceso cognitivo-motivacional orientado a objetivos, más que como un estado emocional aislado^(3,7,8,12)^.

La evidencia analizada sostiene que la esperanza es la capacidad de movilizar la motivación para la consecución de objetivos significativos y, al mismo tiempo, de identificar posibilidades concretas para alcanzarlos, incluso en contextos de adversidad^(7,8,12)^.

Esta conceptualización se ve corroborada por resultados empíricos que demuestran asociaciones consistentes entre niveles más elevados de esperanza e indicadores positivos de adaptación psicológica, en particular la autoestima, el sentido de valor personal y el significado atribuido a la experiencia vivida, lo que refuerza el papel de la esperanza como recurso adaptativo central^(3,7,8)^.

En el conjunto de los estudios analizados, la esperanza adquiere una expresión fuertemente relacional y contextual, y se ve influenciada por factores que pueden, al mismo tiempo, favorecerla o debilitarla^(3,8,9,10,13)^.

El acceso a información clara, comprensible y oportuna sobre el diagnóstico, la evolución de la afección y los recursos disponibles surge de manera consistente como un factor que facilita la esperanza, ya que permite anticipar cambios, manejar las expectativas y reducir la incertidumbre asociada al futuro^(3,8,9,13)^.

Del mismo modo, la presencia de redes de apoyo social significativas —que incluyen a personas en situaciones similares, profesionales y entornos de formación o rehabilitación— se asocia con niveles más altos de esperanza, ya que actúa como facilitadora de los mecanismos de *coping* y de la adaptación psicosocial^(3,8,10,13)^.

Por el contrario, los contextos caracterizados por la desinformación, la falta de acompañamiento, la estigmatización o las actitudes sociales negativas se asocian con el debilitamiento de la esperanza, acentuando los sentimientos de incertidumbre, aislamiento y pérdida de control^(3,10,13)^.

En el contexto específico de la pérdida irreversible de la visión en edad activa, la esperanza surge como un fenómeno central en el proceso de adaptación, configurándose como un recurso adaptativo relacional y procesual^(7,8,9,10,12)^.

En este marco, la esperanza está relacionada con la autoestima y el sentido de valor personal, con la percepción de la capacidad para reorganizar objetivos y rutinas, con la construcción de significado ante la pérdida de la visión (incluida la reconstrucción de la identidad y la reconfiguración de roles socialmente valorados) y con el apoyo social percibido^(7,8,10,12)^.

Así, la esperanza no se manifiesta como la negación de la irreversibilidad de la condición, sino como la capacidad de reorganizar proyectos de vida, encontrar soluciones posibles y atribuir continuidad y sentido a la experiencia, incluso ante limitaciones funcionales progresivas, lo que refleja la naturaleza multidimensional, dinámica y situada de la adaptación psicosocial en este contexto^(3,8,9,10)^.

El análisis de la calidad de vida en contextos de pérdida visual irreversible sugiere que los procesos de adaptación van más allá de la dimensión anatómica o funcional, y surgen principalmente de la forma en que las personas reinterpretan su experiencia vivida, reconstruyen su identidad y redefinen lo que significa vivir bien ante la progresión de la afección. Desde esta perspectiva, la esperanza se manifiesta de manera implícita como una perspectiva de continuidad, de propósito y de valor atribuido a la vida, incluso ante limitaciones funcionales progresivas e irreversibles^(3,9)^.

Los estudios analizados evidencian que la información proporcionada a los pacientes desempeña un papel fundamental en la construcción y el mantenimiento de la esperanza, no limitándose a una función instrumental, sino asumiendo un papel central en la promoción del sentido de control, la previsibilidad y la capacidad de anticipar el futuro. El acceso a información clara, comprensible y adaptada a la persona, particularmente en lo que se refiere al pronóstico, a la progresión de la pérdida de la visión y a los recursos terapéuticos, de rehabilitación y sociales disponibles, surge de manera consistente como un factor que facilita la adaptación psicológica. Esto contribuye a reducir la incertidumbre, el miedo y los sentimientos de desesperanza, además de favorecer procesos más adaptativos de *coping* y reorganización de la vida cotidiana^(3,7,8,9,10)^.

A pesar de la relevancia del fenómeno, la literatura revela lagunas significativas, especialmente en el desarrollo y la evaluación de intervenciones estructuradas que promuevan la esperanza, particularmente en el ámbito de la enfermería. En este sentido, la atención de enfermería integrada en la comunicación terapéutica permite trabajar la esperanza a través de la educación para la salud y el acompañamiento continuo, transformando la información en conocimiento significativo, promoviendo la participación activa de la persona y apoyando procesos de adaptación más autónomos, dignos y psicosocialmente adecuados ante la pérdida irreversible de la visión en edad activa.

### Limitaciones del Estudio

A pesar del rigor metodológico adoptado, esta revisión de alcance presenta algunas limitaciones que es importante reconocer. El reducido número de referencias incluidas en esta revisión refleja la especificidad del fenómeno investigado y la escasez de investigaciones publicadas que aborden de manera directa y sistemática la esperanza en el contexto de la pérdida irreversible de la visión en edad activa, razón por la cual se optó por ampliar el período temporal a los últimos 25 años. Además de la escasez, los estudios disponibles se desarrollaron en contextos heterogéneos en cuanto a la edad, la etiología de la discapacidad visual y el marco sociocultural.

El predominio de enfoques cualitativos y exploratorios, a menudo basados en muestras reducidas, limita la transferibilidad de los resultados. Además, la diversidad conceptual en la definición de la esperanza —a veces descrita como un recurso cognitivo-motivacional, otras como una experiencia existencial o espiritual— dificulta la comparación entre estudios y la consolidación de un marco teórico unificado.

Se observa, asimismo, una escasa exploración de la dimensión temporal de la esperanza, siendo raros los estudios que analizan su evolución a lo largo del proceso de adaptación a la pérdida de la visión. Por último, la ausencia de estudios de intervención limita la extrapolación de los hallazgos a la práctica clínica basada en la evidencia.

### Sugerencias de Mejora e Investigación Futura

Los resultados de esta *revisión de alcance* refuerzan la pertinencia y relevancia del desarrollo de una investigación más profunda sobre el proceso de transición que viven las personas adultas en edad activa ante la pérdida de la visión. La articulación entre la esperanza, el significado atribuido a la experiencia, las estrategias de *coping*, la reconstrucción de la identidad y el apoyo relacional, identificada en la literatura analizada, apunta a la necesidad de comprender este fenómeno de manera procesual y contextualizada, integrando las dimensiones biológica, psicológica, social y espiritual de la experiencia vivida. Estos hallazgos respaldan la elección de un enfoque cualitativo de naturaleza interpretativa, como la Teoría Fundada en los Datos Constructivista, como una metodología capaz de captar la complejidad del proceso de transición y de dar voz a las personas en la construcción de los significados asociados a la pérdida de la visión.

Los estudios futuros también deberán dar prioridad a los diseños longitudinales, que permitan comprender la evolución de la esperanza desde el diagnóstico hasta las fases más avanzadas de adaptación y reconstrucción de la identidad, profundizando en la investigación sobre la esperanza como proceso dinámico en el contexto de la pérdida irreversible de la visión.

También se recomienda la validación de instrumentos sensibles a la experiencia subjetiva de la esperanza en personas con discapacidad visual, lo cual constituye otra laguna relevante.

En el ámbito de la práctica clínica, se necesitan estudios de intervención que evalúen estrategias estructuradas para promover la esperanza, mediante programas educativos, acompañamiento de enfermería enfocado en la transición y la facilitación del acceso a la información y a las redes de apoyo, con el objetivo de brindar una atención más humanizada y centrada en el paciente^(3,7,8,9,10,11,12)^.

## CONCLUSIÓN

Esta revisión de alcance pone de manifiesto, de manera consistente, que la esperanza es un recurso adaptativo central en el proceso de transición que viven las personas en edad activa ante la pérdida irreversible de la visión.

Los resultados demuestran que la esperanza se configura como un fenómeno dinámico, relacional y contextual, estrechamente vinculado a la autoestima, el significado personal, el apoyo social y el acceso a información clara y relevante, y que desempeña un papel determinante en la adaptación psicosocial, la reorganización de los proyectos de vida y la reconstrucción de la identidad.

A pesar de estas aportaciones, el análisis de la literatura revela una producción científica aún limitada y fragmentada, caracterizada por el predominio de enfoques exploratorios, la escasez de estudios longitudinales y la inexistencia de intervenciones estructuradas orientadas a la promoción de la esperanza, particularmente en el ámbito de la enfermería, lo que refuerza la necesidad de profundizar en la investigación y desarrollar modelos conceptuales integradores que sustenten prácticas clínicas basadas en la evidencia.

En este contexto, la enfermería surge como ciencia en la mediación de la esperanza, a través de prácticas centradas en la persona que valoran la comunicación terapéutica, la educación para la salud, el acompañamiento a lo largo de la transición y la facilitación del acceso a recursos y redes de apoyo, contribuyendo a una atención más humanizada, integrada y sensible a la experiencia vivida, con impacto en la dignidad, el bienestar y la calidad de vida de estas personas.

## Data Availability

Los datos están disponibles en: https://osf.io/gcxev.
